# Cracking the code of teacher burnout: the chain mediation of GPT integration degree through behavioral engagement and classroom atmosphere in a cross-level chain mediation model

**DOI:** 10.3389/fpsyg.2024.1495743

**Published:** 2024-11-13

**Authors:** Bingyue Chen, Binglian Chen, Shengtao Ren, Bin Li, Hui Liu, Guoxin Jiang

**Affiliations:** ^1^Taizhou Vocational College of Science and Technology, Taizhou, China; ^2^Zhejiang Gongshang University, Hangzhou, Zhejiang, China

**Keywords:** chat GPT, behavioral engagement, classroom atmosphere, GPT integration degree, teacher burnout

## Abstract

Chat GPT technology plays a pivotal role in global educational innovation and the enhancement of the quality of teaching and learning. In the field of education research, numerous studies have been conducted to investigate the effectiveness of GPT technology, teacher acceptance, and student engagement in depth. To date, few studies have considered the compounding effects of these factors on teacher burnout from the perspectives of psychology and behavioral sciences in conjunction with the dichotomous and complex relationship between teachers and students. Consequently, based on the findings of previous scholars, this study constructed a cross-layer chain mediation model based on the SOR and EASI models. This model was used to explore how different motivators affect the alleviation of teacher burnout through psychological and behavioral mechanisms. The study involved 47 teachers and 506 students from 10 universities. The findings of the study indicated that (1) the direct effect of GPT integration degree on teacher burnout was not statistically significant, and (2) the classroom atmosphere played a pivotal mediating role in the relationship between GPT integration degree and teacher burnout. (3) The degree of GPT integration degree exerts an indirect and orderly negative influence on teacher burnout through behavioral engagement and classroom atmosphere. The objective of this study is to further enhance our comprehension of the utilization of GPT technology in education and to provide strategic recommendations for its advancement in educational practice.

## 1 Introduction

On November 30, 2022, OpenAI, a U.S. artificial intelligence research laboratory, released its newest chatbot model, Chat GPT. This immediately caused a strong “surprise effect” in the tech and academic worlds, and was rapidly adopted in the Internet sector with more than 100 million users. Chat GPT has transformed the manner in which we interact with AI, and its exceptional capabilities have also had a profound impact on the education sector, with the potential to drive significant shifts in educational concepts and practices. Chat GPT’s language model assists students in a variety of ways, such as providing information resources, helping to improve language skills, improving learning efficiency (Fauzi et al., 2023), and There are also untapped potential benefits in streamlining enrollment, improving student services, enhancing instruction, research assistance, and increasing student retention (Dempere et al., 2023). At present, the integration of Chat GPT with the education field has achieved fruitful practical results: educators try to use GPT for personalized tutoring, content generation and intelligent feedback mechanisms to help students learn according to their own learning needs (Jafarzade, 2023). In language learning, some students preparing for IELTS and TOEFL exams use GPT to simulate real conversation scenarios and interact with the models to practice grammar, expand vocabulary and improve fluency. At the same time, GPT has a superior role to play in facilitating the transformation of educational practices (Grassini, 2023); supporting the potential of self-determined learning (Baskara, 2023), and so on.

The extensive application of GPT technology in the field of education has led to a situation in which teachers, as core participants and irreplaceable players in the educational process, benefit from the convenience offered by educational innovations. At the same time, however, they are on the front line of a profound transformation of the educational model. In the face of profound changes to the teaching model, an ever-increasing teaching load, and the accumulation of psychological pressure, teachers are also subjected to the impact of technological change, which can lead to a deep sense of burnout. In light of these developments, it has become increasingly clear that teacher burnout represents a significant issue in the field of education that cannot be overlooked. Teacher burnout is characterized by three main symptoms: extreme fatigue, indifference to work, and decreased self-efficacy. These symptoms not only cause teachers to suffer personally but also affect their enthusiasm and efficiency in the classroom. This, in turn, affects students’ interest in learning and academic achievement (Oliveira et al., 2021). Given the significant impact of teacher burnout on teachers’ psychological well-being and professional satisfaction, as well as its direct negative effect on the quality of education and student learning outcomes, it is crucial to gain a deeper understanding of this phenomenon and develop effective responses to improve the overall performance of the education system. In response, academics have initiated research on the topic with great alacrity, as educators and researchers have begun to focus on studies of teacher burnout, exploring ways to alleviate or even eradicate the problem. These explorations included the implementation of strategies such as the expansion of professional development opportunities for teachers and the provision of mental health support. However, these solutions are often characterized by fragmentation and a lack of systematicity, and fail to demonstrate sufficient foresight and adaptability when considering new changes triggered by technological advances (Appendix Table 1).

During the data survey phase, it was discovered that there are already teachers who are able to use Chat GPT’s various plug-ins in a flexible manner to assist in the creation of lesson planning PowerPoints as well as the writing of lesson plans. According to an anonymous respondent, “Since we are afraid of AI replacing us, we might as well learn to take control of them.” In light of the rapid advancement of AI technologies such as GPT modeling, it is reasonable to posit that the convergence of technologies may open up new avenues and strategies for mitigating teacher burnout. Although research in this area is still in its infancy, there is a notable absence of systematic exploration into the impact of GPT technology integration on alleviating teacher burnout. Against this background, it is an urgent and unanswered question to explore whether and how GPT technology can effectively reduce teacher burnout and improve the quality of education accordingly.

Existing research on the impact of Chat GPT and others on the education sector often focuses only on the teacher side alone (Montenegro-Rueda et al., 2023) or the student side alone (Thong et al., 2023), with little research perspective that encompasses both the teacher and student sides. The Cross-level Chained Mediation model is one of the few models in the field of quantitative social sciences that can quantify the bi-lateral aspects, and it is also a mediation model that exchanges data or information in multi-layer architectures, which can effectively communicate and coordinate between different systems or network layers. In contrast to traditional mediation models that typically operate within a single layer, with each layer handling only the information directly relevant to it, the cross-layer chained mediation model allows information to be passed directly between different layers (Chen et al., 2024). Thanks to the advantages of cross-layer chain mediation, this paper breaks the limitations of traditional SEM and constructs a cross-layer chain mediation model based on the perspectives of both teachers-students to provide new kinetic insights into the link between GPT integration degree and teacher burnout, and to provide suggestions for educational practitioners and administrators with a fresh perspective.

## 2 Literature review and hypotheses

### 2.1 GPT integration degree and teacher burnout

With the publication of the first research article on burnout, the concept was formally recognized in psychology as a field. Burnout was initially described as the state of exhaustion among workers in helping professions (Schwab, 1996). As researchers delved deeper into burnout across various fields, a theoretical framework emerged. Chemiss, representing the organizational perspective, systematically analyzed the impact of environmental factors on burnout through an organizational science lens (Pines, 1993), attributing the main influencing factors to organizational and socio-cultural backgrounds. On the other hand, Maslach and her colleagues categorized burnout into three dimensions—emotional exhaustion, depersonalization, and low achievement—from a social psychological standpoint (Maslach et al., 1996), developing a burnout assessment questionnaire based on these dimensions. Researchers have expanded their study of burnout beyond the clinical realm to encompass social history, organizational science, and social psychology. Despite these varied perspectives, they can be broadly summarized into two points: first, burnout arises within specific contexts, including high work intensity and self-expectations; second, burnout is a negative individual state, manifested in symptoms such as emotional exhaustion, a sense of loss, and low achievement. These symptoms not only affect the physical and mental health of teachers and the quality of their work but also indirectly impact students’ academic performance and overall development.

In the realm of software and technology, “integration” refers to the extent to which a particular technology or software is harmonized with existing systems. In environmental science, “integration” may refer to the extent to which a specific policy or practice is integrated within a broader environmental management system (Persson and Gerger Swartling, 2002). Based on our literature review, the term “GPT integration degree” lacks a clear and consistent definition in academia, with differing opinions among experts and scholars. Fadel considers GPT integration degree as its potential application in education, discussing the benefits and challenges of integrating AI technologies (Holmes et al., 2019), while Zawacki-Richter’s systematic review evaluates Artificial Intelligence in Higher Education, providing a comprehensive view of AI technologies, including GPT integration degree and practical effects (Zawacki-Richter et al., 2019). Based on this, this paper defines GPT integration degree as the proficiency of an individual or organization in understanding and utilizing generative pre-trained transformer (GPT) models, encompassing their broad application capabilities and technical depth. This includes a comprehensive understanding of how GPT functions, including its capabilities and limitations, and how to apply this technology across various scenarios, such as text generation, information retrieval, and automated dialog. It also includes innovative applications of GPT in non-traditional contexts, proficiency in using relevant plugins and tools, and the ability to provide flexible instructions to GPT models based on specific information needs, among other things.

Classroom teaching is the primary responsibility of teachers, and the application of new technology such as GPT to education aims to enhance the efficiency of education and teaching, reduce teachers’ workload, and ultimately decrease burnout. Jacques suggests a close relationship between technology and efficiency (Ellul, 2018), highlighting that the essence of technology lies in the pursuit of efficiency. GPT, as a new type of technological intelligent agent, can effectively lessen the burden on teachers’ time, physical and cognitive stamina, and emotions. This reduction in workload can enhance teachers’ work and teaching efficiency in both the classroom and after-school activities, reflecting in their behavior, discourse, and professional relationships. GPT can be utilized not only in classroom teaching but also in after-school feedback and teaching evaluation, greatly reducing teachers’ repetitive and mundane tasks and, consequently, reducing their sense of burnout (Hakanen et al., 2006; Chaudhry and Kazim, 2022; Ming, 2005). Additionally, using GPT as an AI tool allows for qualitative assessment of students, further easing teachers’ workload (Samarakou et al., 2014). GPT can swiftly and accurately complete evaluations, improving efficiency and overcoming the shortcomings of traditional manual evaluation methods, which are time-consuming and labor-intensive. Moreover, GPT can objectively evaluate the quality of teachers’ teaching through big data analysis, providing teachers with a more objective understanding of their teaching level and avoiding the influence of personal bias on evaluation results. This can help prevent personal negative emotions from affecting the teaching environment, which can lead to harmful effects and burnout. In conclusion, GPT plays a crucial role in reducing teachers’ workload, fostering teaching innovation, enhancing teachers’ enthusiasm for teaching, and improving teaching quality, ultimately reducing burnout. GPT, as a new technological innovation, can be applied in teachers’ daily teaching environments to reduce their time, physical strain, cognitive load, and emotional stress, thus alleviating burnout. Based on this, this paper proposes the following hypothesis:

*H1*: GPT integration degree has a negative inhibitory effect on teacher burnout.

### 2.2 Behavioral engagement, GPT integration degree, and teacher burnout

Engagement refers to a positive state exhibited by individuals in all aspects of mind and body during an activity. Behavioral engagement, specifically in educational settings, pertains to students’ interactions and responses, including adherence to rules, and active involvement in academic and extracurricular activities (Ladd and Dinella, 2009). It is considered a prerequisite for good academic performance (Fredricks et al., 2004). A high level of behavioral engagement can enhance students’ motivation to learn, increase their classroom attention, and improve their academic performance (Reschly and Christenson, 2012). In this paper, behavioral engagement is defined as the psychological state and behavioral tendency that students exhibit in activities. It significantly influences teachers’ psychological state during classroom interactions. When students show enthusiasm and interest in learning, teachers perceive their teaching as effective, receiving positive feedback. This feedback enhances teachers’ professional self-esteem and satisfaction, reducing burnout and improving students’ academic achievement (Maslach and Jackson, 1981). Highly engaged students facilitate a smoother teaching process, reducing the pressure on teachers to manage the classroom, allowing them to focus more on content deepening and innovation (Skinner and Belmont, 1993). Conversely, a lack of student engagement may lead teachers to question their teaching abilities and career choices, contributing to emotional exhaustion and burnout. As Friedman discusses, low student engagement can cause teachers to feel conflict and frustration in their professional roles (Friedman, 1995; Covell et al., 2009).

The theory of embodied behavior emphasizes that an individual’s cognition and behavior are shaped through direct interaction with the social environment (Glenberg, 2010). In daily classrooms, students can enhance their participation and embodied experiences through interactive teaching methods, such as hands-on, demonstrative, and experiential learning activities. Such engagement not only stimulates students’ enthusiasm and curiosity, improving learning outcomes, but also increases teachers’ professional satisfaction and motivation, fostering a positive teacher-student relationship, and reducing burnout (Lindgren and Johnson-Glenberg, 2013). Thus, this paper concludes that students’ behavioral engagement significantly affects teacher burnout, with high engagement levels leading to reduced burnout. Effective mastery and application of GPT technology in teaching can impact students’ learning status. It enhances classroom interaction by enabling teachers to provide targeted content, meeting students’ needs, and increasing interactivity (Holmes et al., 2019). Additionally, GPT provides real-time feedback according to students’ learning progress and performance, allowing for independent learning while providing specific behavioral feedback to teachers. This feedback loop enables teachers to adjust teaching strategies in real time (Baker, 2016). Based on this, the paper proposes the following hypothesis:

*H2*: Behavioral engagement mediates the relationship between GPT integration degree and teacher burnout.

### 2.3 Classroom atmosphere, GPT integration degree, and teacher burnout

Classroom atmosphere encompasses all the elements within the classroom environment that students can sense and which influence their learning experiences. A positive classroom atmosphere is a key indicator of effective teaching, and assessing this atmosphere can serve as a valuable tool for evaluating classroom quality. Educational research consistently shows that a supportive and engaging classroom atmosphere can boost student involvement, ignite their motivation to learn, and significantly improve educational outcomes (Yan-chu, 2013; Shernoff et al., 2017). For instance, Weinstein’s research indicates that classroom atmosphere is influenced not just by the physical arrangement and visual appeal of the classroom, but also by the interactions between teachers and students, as well as the emotional expressions observed during the teaching process. These factors collectively impact students’ emotional well-being and their motivation to learn (Weinstein, 1991). This paper provides a summary of recent studies, dividing classroom atmosphere factors into four distinct categories: physical environment factors (Oliveras-Ortiz et al., 2021), teacher-student interaction factors (Luo et al., 2022), teaching strategies factors (Li and Xue, 2023) and technology application factors. This paper concentrates on the technology application factor: As educational technology advances, integrating technology into the classroom environment has emerged as a new area influencing student engagement. Research demonstrates that employing technology tools, such as smart classroom technology and online learning platforms, can improve the classroom atmosphere, particularly in distance learning settings (Luo et al., 2022). Moreover, the manner in which teachers utilize technology for pedagogical innovation is equally vital. Effective use of technology can enhance both cognitive and affective student engagement. The synergy of technology and interactivity in the classroom plays a crucial role. Learning outcomes can be significantly improved by optimizing classroom design, incorporating modern technology, and enhancing teacher-student interactions.

In recent years, the increasing use of AI technologies, particularly GPT, in education has prompted concerns about their impact on the teaching environment and teachers’ professional well-being. AI tools like GPT are credited with optimizing the teaching and learning process, reducing teachers’ daily instructional burdens, and lessening their workload through automated content generation and management. However, the classroom atmosphere, which encompasses student engagement, the quality of teacher-student interactions, and the effective use of teaching resources, plays a critical role in influencing teacher-student dynamics, student motivation, and teachers’ job satisfaction and burnout. Optimizing the classroom atmosphere not only enhances teaching effectiveness but also alleviates the pressures teachers face. A dynamic classroom environment directly boosts student engagement. A lively and inclusive classroom atmosphere fosters students’ interest and motivation, encouraging more active participation in classroom activities. Moreover, the implementation of GPT tools in the classroom, such as real-time interactive quizzes and personalized learning suggestions, has the potential to significantly enhance student engagement. This, in turn, can reduce the demands on teachers to maintain classroom order and boost student involvement.

This paper outlines four specific real-life impacts of using GPT in education: First, it enhances teaching effectiveness and teacher satisfaction. Teachers who utilize GPT can significantly improve the classroom atmosphere by providing personalized learning experiences and boosting student engagement. Second, it helps alleviate teachers’ emotional exhaustion. For instance, REEM’s study investigated the efficacy of Chat GPT as a teacher’s assistant in reducing workload and preventing burnout. The findings suggest that incorporating AI tools in the classroom can optimize teachers’ planning and resource allocation, potentially reducing job stress and burnout (Hashem et al., 2024). Third, it fosters positive teacher-student interactions. Chang et al.’s research demonstrated that teachers’ cognitive assessments of student behaviors and their strategies for regulating emotions are crucial in managing burnout (Chang, 2013). GPT tools can assist teachers in better responding to students’ needs and interests by generating customized feedback and interactive content. This enhanced interaction not only improves students’ learning experiences but also boosts teachers’ pedagogical satisfaction and reduces burnout. Fourth, GPT facilitates the optimization of teaching resources. For instance, it can be used to create interactive courseware and simulation experiments, enriching the teaching content. These resources improve classroom dynamics and pedagogical variety, making the environment more engaging and supportive, and reducing the pressure teachers face due to classroom preparation and content updating. In summary, an excellent classroom atmosphere not only fosters student learning but also significantly alleviates teacher stress and burnout. Teachers’ adept use of tools like GPT can further enhance the classroom atmosphere, creating a more productive and satisfying teaching and learning environment for both teachers and students. In light of the aforementioned evidence, the following hypotheses are put forth in this paper:

*H3*: Classroom atmosphere mediates the relationship between GPT integration degree and teacher burnout.

### 2.4 GPT integration degree, behavioral engagement, classroom atmosphere, and teacher burnout

The significance of classroom atmosphere in an educational setting is paramount for the quality of teaching and learning outcomes. A positive classroom atmosphere not only boosts student motivation and academic achievement but also profoundly influences teachers’ professional satisfaction and enthusiasm (Reyes et al., 2012; Dorman, 2003). However, teachers frequently encounter a range of challenges in their careers, including student behavioral issues and overloaded curricula. These challenges can often lead to negative moods and a diminished sense of accomplishment (Hastings and Bham, 2003), which together constitute the core manifestations of teacher burnout. However, if the classroom atmosphere is positive, students are performing well, and teachers effectively manage the classroom, this not only enhances teaching effectiveness but also significantly reduces teacher stress, thereby decreasing the risk of burnout (Conroy et al., 2009). Additionally, an improved classroom atmosphere enhances teacher-student interactions, which further boosts student motivation and engagement in the classroom. This improvement in the educational environment not only elevates teaching quality but also helps in reducing teacher burnout.

GPT has emerged as a pivotal technology in today’s rapidly evolving educational landscape, driving innovation by increasing the accessibility and personalization of educational content. This enhances the overall student learning experience. By offering real-time, interactive learning support, GPT technology can significantly boost students’ behavioral engagement, thereby fostering greater motivation and involvement in the learning process (Adıgüzel et al., 2023; Rejeb et al., 2024). Embodied cognition theory highlights the significant role of physical state and environmental context in shaping cognitive processes, suggesting that learning and cognition extend beyond the brain to interactions with the physical environment. Applying this theory to assess the educational impact of GPT technology offers a deeper insight into its benefits on student behavioral engagement, classroom atmosphere, and teacher burnout. Specifically, GPT technology enhances students’ interactive experiences, aligning with the “experiential learning” concept from embodied cognition theory. The personalized feedback and interactive simulations provided by GPT enable students to acquire knowledge through concrete actions and experiences. This approach emphasizes the role of physical actions and sensory experiences in enhancing cognitive processes, thereby enriching the learning environment and effectiveness (Hashem et al., 2024; Wang et al., 2015). Second, as students achieve higher levels of behavioral engagement through GPT technology, their physical and emotional engagement also intensifies. This heightened engagement directly enhances the classroom atmosphere. According to embodied cognition theory, physical and emotional states can significantly influence cognitive states. The active participation of students energizes the entire classroom environment, creating a dynamic atmosphere that is conducive to improved learning outcomes. In such an environment, students engage with the content not just intellectually but also on emotional and physical levels, which amplifies the learning effects and deepens their understanding (Wang et al., 2015; Conroy et al., 2009). Finally, by enhancing the classroom atmosphere and fostering students’ active participation, GPT technology indirectly alleviates the teaching pressures faced by educators. Embodied cognition theory posits that teachers’ cognitive and emotional states are influenced by the physical environment. A positive classroom environment can lessen teachers’ physical and psychological burdens, allowing them to feel more relaxed and content. This reduction in stress helps mitigate teacher burnout, enabling educators to engage in teaching activities with more positive attitudes. This not only elevates the quality of teaching but also boosts teachers’ overall job satisfaction, creating a more fulfilling and effective educational setting (Amin, 2023). Therefore, enhancing students’ behavioral engagement through the application of advanced technologies like GPT not only improves students’ learning outcomes but also positively impacts the entire educational ecosystem by enhancing teaching efficiency and teachers’ job satisfaction. Based on these insights, the following hypotheses are proposed in this paper:

*H4*: GPT integration degree indirectly ordered to negatively influence teacher burnout through behavioral engagement and classroom atmosphere.

## 3 Study design

### 3.1 Sample and data collection

The research data in this paper comes from students and teachers of 10 universities, including Taizhou Institute of Vocational Technology, Zhejiang Gongshang University, and Xi’an Jiaotong University in China, covering a wide range of different majors in literature, engineering, and science. The selection of schools includes colleges and universities in eastern, central and western China, which can reflect the characteristics of teaching time limits in different regions. Before distributing the questionnaires, the researchers conducted short interviews with university teachers to determine the new changes brought by Chat GPT technology to teaching and learning in universities. In order to minimize bias, the questionnaire was distributed in three stages and all data were collected under the guidance of the researcher. The questionnaires returned at each stage were utilized to exclude outlier cases using SPSS 26. 0. In the first stage, teacher burnout was first assessed. A total of 103 questionnaires were distributed and 96 questionnaires were returned with a return rate of 93.2%; in the second phase, one week after the first questionnaire was distributed, the researcher distributed another questionnaire on GPT integration degree to 557 students (The group of students studied were the students taught by the group of teachers in the first phase, in other words, the sample of students in the second phase were taught by the sample of teachers in the first phase), and this time 524 valid questionnaires were returned with a validity rate of 94.1%; in the third phase, one week after the second questionnaire was distributed, 524 of the students who responded effectively, a questionnaire on behavioral engagement and classroom atmosphere was distributed, returning 506 valid questionnaires with a validity rate of 96.6%. Therefore, this paper uses survey data from 47 teachers with 506 students as the sample for analysis. Because this paper’s research strictly follows the teacher-student correspondence, the sample collection process meets the needs of cross-level analysis for the nested nature of the data. In terms of sample size, the N: q rule can be used to roughly determine the required sample size. Where N is the sample size and *p* is the parameter to be estimated in the model, the recommended ratio is 20:1, which can be relaxed to 10:1. In this study, a total of 22 variable measurement items were finally determined, and there were about 45 parameters to be estimated. Therefore, the sample size of 47 + 506 in this article is greater than the required sample size, and the sample size in this article is of analytical significance.

### 3.2 Measurement of variables

Each of the main variables addressed in this paper were drawn from and measured using established and proven scales developed by previous scholars. As a cross-level analysis, this study employed different data collection methods at the teacher level and student level, respectively. This article adopts the “two-way translation” procedure. Two graduate students and two undergraduate students in education independently translated the English version of the scale into Chinese. After summarizing and discussing, a first draft was determined. Then, two English experts translated the Chinese draft back into English, comparing sentence by sentence the degree of consistency between the Chinese translation and the original English questionnaire. Finally, after repeated comparisons and discussions with education experts, it was confirmed as a measurement tool. The variables were measured on a 5-point Likert-typed scale, where 1 means “totally disagree or totally disagree” and 5 means “totally agree or totally agree.”

This paper’s measure of teachers’ Ghat GPT integration degree draws on the scales used by Kerckaert et al. (2015), Compeau and Higgins (1995), and Davis (1989) in their previous studies of teachers’ use of information technology, and this paper focuses on the attitudes toward technology use and technology self-efficacy dimensions of the previous studies, culminating in the 5-item questionnaire of this paper, such as “In the next time, I do not think I will give up using Chat GPT technology in my teaching as an aid.” This paper uses the School Engagement Scale developed by Fredricks et al. (2004) to measure the degree of students’ behavioral engagement, on the basis of which appropriate changes are made according to the characteristics of the research subjects in this paper, and finally forms a quasi 5-item questionnaire on students’ behavioral engagement, such as “Teachers using Chat GPT technology to assist in teaching in the classroom will make me feel fresher”.

Most of the research on classroom atmosphere is based on the variable of learning atmosphere, based on this, this paper draws on the “Adolescents’ Perceived School Atmosphere Scale” compiled by Jia et al. (2009). The scale includes three dimensions of teacher support, peer support, and opportunities for autonomy, and this paper is based on the teacher support part of the three dimensions matching the content of this paper, and modifies part of the expression by adding some Chat GPT has brought new changes to classroom teaching, and finally formed a four-question questionnaire for classroom atmosphere, such as “Watching the teacher’s new form of GPT courseware makes me enjoy discussions with my classmates more.” In this paper, we draw on the MBI Educator Survey (MBI-ES), a scale developed by Maslach (1981) and Maslach et al. (1996) that is specifically designed to test for teacher burnout, to measure teacher burnout. The scale is now widely used in burnout correlation studies and has good cross-cultural reliability and validity. The scale contains three dimensions of emotional exhaustion, depersonalization, and low personal accomplishment, with a total of 22 questions. Among them, there are 9 questions in the emotional exhaustion dimension, 5 questions in the depersonalization dimension, and 8 questions in the low personal accomplishment dimension, all of which are based on a 5-point Likert scale. On this basis, this paper also draws on the question-asking method of the BM scale compiled by Pines and Aronson (1988), which is applicable to different occupational groups, with a total of 21 question items containing three aspects of burnout in the individual’s physiological, emotional, and mental aspects, and each subscale has 7 question items, with the higher the score, the higher the level of burnout in the individual. In this paper, the scale is streamlined and processed to finally form the 8-item scale of teacher burnout in this paper, such as “I often have a sense of frustration in my teaching work.”

## 4 Data analysis and results

### 4.1 Data aggregation test

The intra-team member consistency coefficient Rwg, the within-group degree of difference value ICC (1), and the between-group difference value ICC (2) were used as indicators of data aggregation tests at the teacher and student levels. The Rwg mean, Rwg median, ICC (1), and ICC (2) for each variable were GPTID (0.809, 0.915, 0.589, and 0.939), BP (0.899, 0.922, 0.662, and 0.955), and CA (0.840, 0.904, 0.596, and 0.941), respectively. The Rwg median, the mean, and the ICC were all were higher than the recommended value of 0.7, and both ICC (1) and ICC (2) were also greater than the critical values of 0.12 and 0.7, respectively. Therefore, the student-level data can be aggregated and averaged to aggregate to the classroom level.

### 4.2 Analysis of common method bias and validation factors

In order to avoid common methodological biases, this paper measured independent and dependent variables from different sources and at different time points, and a precautionary treatment was done beforehand to reduce survey respondents’ motivation to fill in the questionnaire in a consistent manner. However, because the questionnaire is self-assessed and subjective, the possibility of homogeneity bias still exists. Therefore, this paper conducted a Harman one-way test, drawing on the method of Podsakoff and Organ (1986). The results show that there is more than one factor with an eigenroot greater than 1 and none of the factors has a variance explained greater than 40%, which suggests that there is no serious problem of homophily bias in this paper.

Among the variables addressed in this paper, GPT integration degree and teacher burnout are high-level (between-groups) variables, and behavioral engagement and classroom atmosphere are covariant at both low (within-groups) and high (between-groups) levels. According to previous studies, it is possible that scores on different dimensions of a high-level variable, which serve as measures of latent variables, are covariant, due to the fact that their covariances may originate from other antecedent variables. Therefore, this paper applies the multilevel validated factor analysis (MCFA) method to explain the group-level effects while validating the individual level. The MCFA results show that the hypothesized model has a good fit ( χ 2 = 76.94, df = 53, CFI = 0.982, TLI = 0. 975, RMSEA = 0.03, SRMR (within-group) = 0.04, and SRMR (between-group) = 0. 064), and all indicators of latent variables were significant. Therefore, the scale measures in this paper have good discriminant validity and the hypothesized model is supported.

### 4.3 Descriptive statistics of variables, correlation analysis and reliability and validity tests

**Figure 1:**
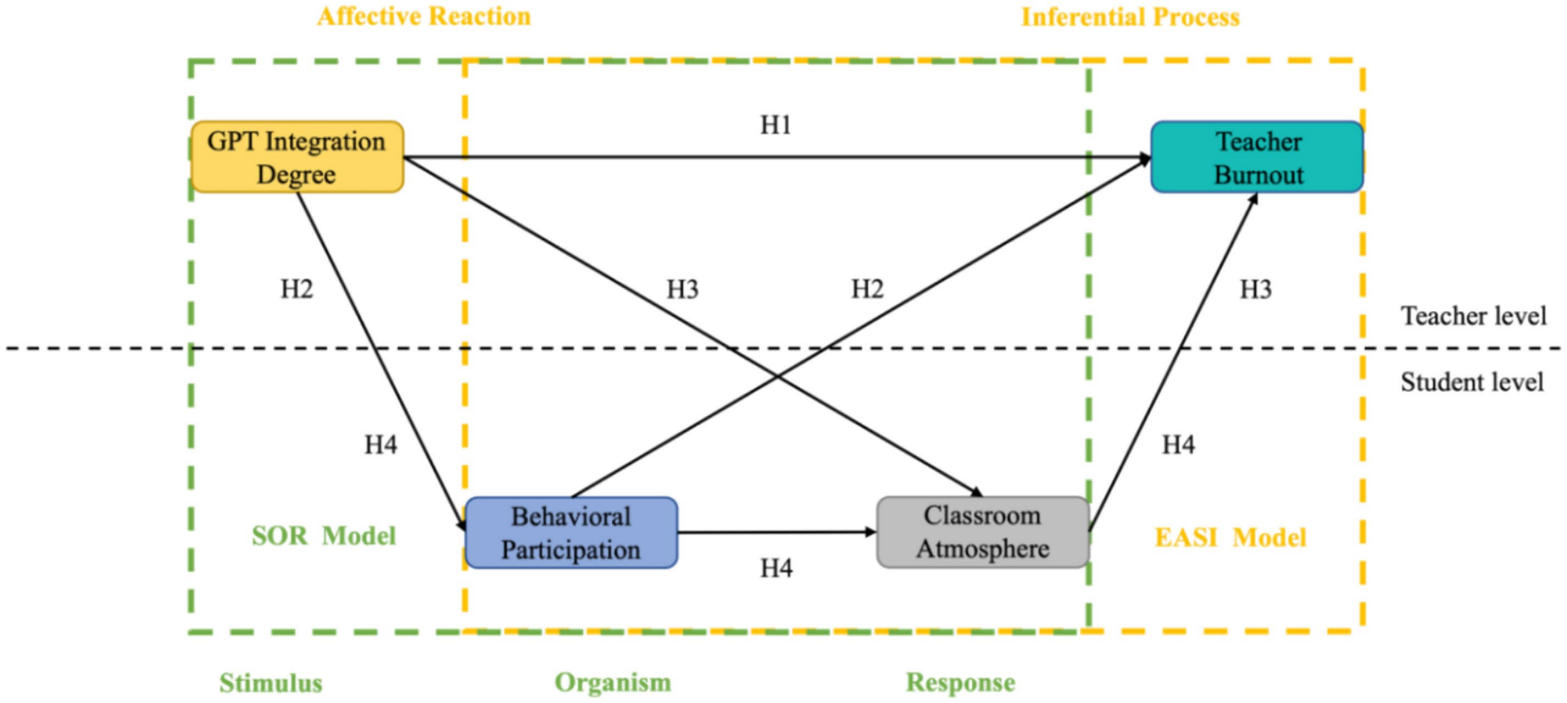
Proposed model diagram.

Table 1 presents the results of descriptive statistics and correlation coefficients for each of the study variables, reflecting the mean, standard deviation, and correlation coefficient matrices for student-level and teacher-level variables. The results of the consistency test for each variable showed that the Cronbach’s alpha values for GPT integration degree, behavioral engagement, classroom atmosphere, and teacher burnout were 0.900, 0.894, 0.868, and 0.927, respectively. and the joint reliabilities (CR values) were greater than 0.7 (see Table 2 for more details), indicating that each scale in the present study had good reliability (Figure 1).

**Table 1:** Variable means, standard deviations, and pearson correlation.

	Average value	Standard deviation	BP	LC	GPTA	TB
Personal level						
BP	3.311	0.952	1			
CA	3.239	0.964	0.605**	1		
Team level						
BP	3.311	0.79	1			
CA	3.239	0.764	0.865**	1		
GPTID	3.329	0.746	0.508**	0.521**	1	
TB	3.033	1.018	−0.496**	−0.453**	−0.223**	1

*p < 0.05, **p < 0.01, **p < 0.001.

**Table 2:** Results of model confidence, AVE, and CR metrics.

Factor	Cronbach’s alpha coefficient	Mean variance extraction AVE value	Combined reliability CR
GPTID	0.900	0.643	0.900
BP	0.894	0.629	0.894
CA	0.868	0.622	0.868
TB	0.927	0.615	0.927

Validated factor analysis was conducted using Mplus 8.3 software and the results showed that the factor loadings of all the items reached the level of 0.7 or more and were significant at the 0.001 level of significance, which indicates that there is statistical significance between the indicators and the variables being measured. The AVE estimates of the four variables were calculated to be higher than the squared value of their correlation coefficients, and the measurements of the constructs in this study satisfy convergent validity and have good discriminant validity.

### 4.4 Analysis of cross-layer chain mediation effects

**Figure 2:**
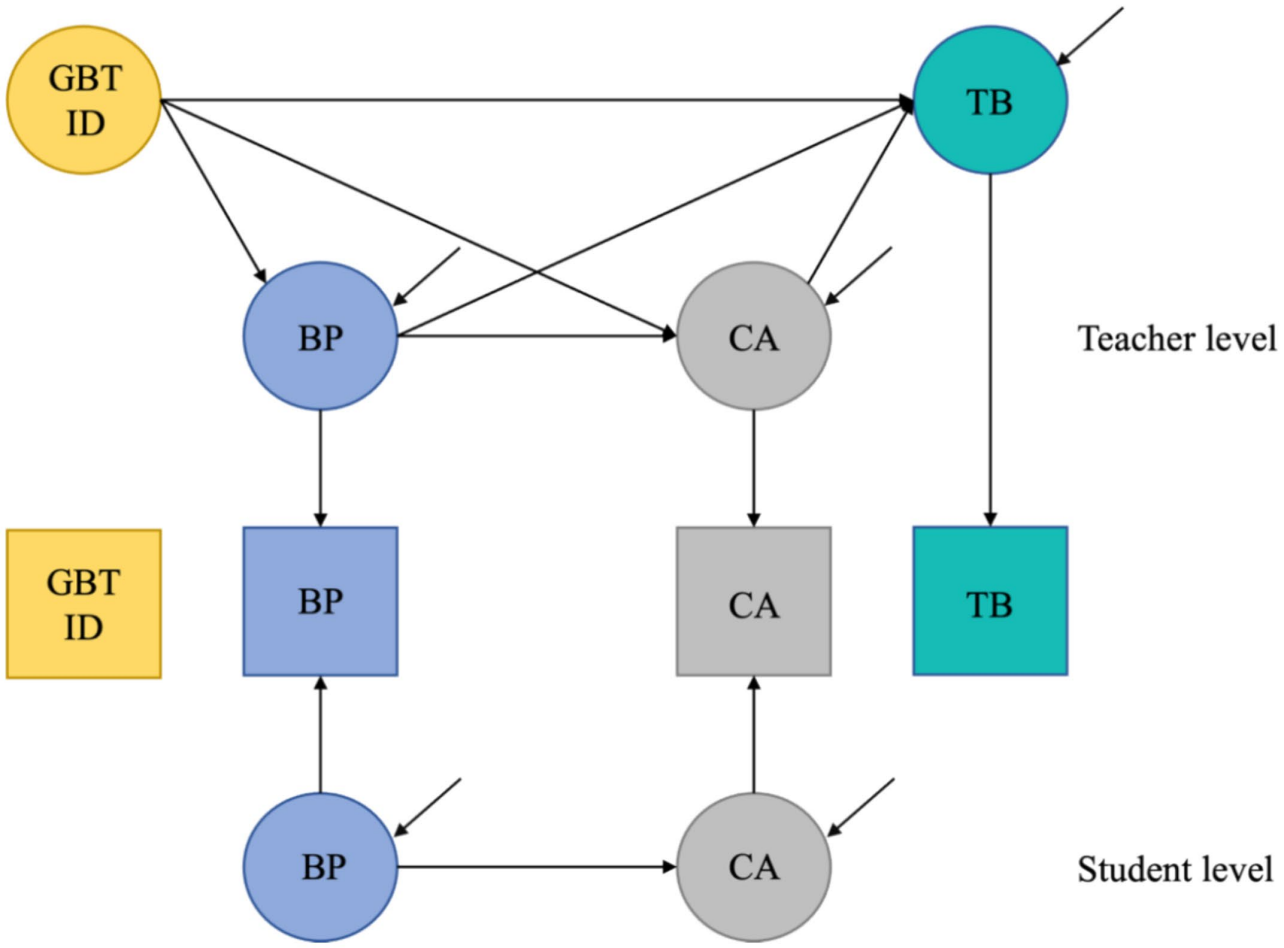
Cross-layer chain mediation between variables.

The relationships between the independent variables, mediator variables, and dependent variables at different levels of the hypothesized model are shown in Figure 2. The MSEM can simultaneously estimate: the “top-down” influence relationship between teachers’ GPT integration degree and student behavioral engagement and classroom atmosphere; the influence relationship between student behavioral engagement and classroom atmosphere at different levels (student level and teacher level); and the “bottom-up” influence relationship between student behavioral engagement, classroom atmosphere and teacher burnout. “Bottom-up” relationships between student behavioral engagement, classroom atmosphere, and teacher burnout.

This study follows the recommendation of Preacher et al. (2010) to examine the chain relationship between latent variables and latent group means at the between-group level. In this paper, we use Mplus 8.3 software to apply Robust Maximum Likelihood (MLR) method to analyze the data for multilevel path analysis, which tests multiple paths while taking full account of the nested nature of the data, and the MLR method has strong utility for multilevel mediator analysis (Preacher et al., 2011). In this paper, the potential predictors, the potential group means of the two mediators and the three path coefficients between the potential outcome variables are multiplied to calculate the chain mediation effect, and the point estimates and confidence intervals of this chain mediation effect are obtained based on the unstandardized coefficients, and the model test results are shown in Table 3.

**Table 3:** MSEM analysis results.

	Efficiency value	SE	*p*	95% confidence Interval
GPTID→BP	0.471	0.139	0.001	[0.199, 0.743]
GPTID→CA	0.382	0.142	0.007	[0.104, 0.660]
BP → CA (within group)	0.170	0.076	0.025	[0.021, 0.319]
BP → CA (intergroup)	0.480	0.132	0.000	[0.220, 0.739]
GPTID→TB	−0.042	0.114	0.712	[−0.266, 0.182]
BP → TB	−0.455	0.149	0.002	[−0.746, −0.164]
CA → TB	−0.472	0.153	0.002	[−0.772, −0.172]
Indirect relationship test				
GPTID→BP → TB	−0.214	0.087	0.014	[−0.386, −0.043]
GPTID→CA → TB	−0.181	0.06	0.003	[−0.298, −0.063]
GPTID→GP → CA → TB	−0.103	0.046	0.025	[−0.193, −0.013]

As shown in Table 3, GPTID→BP was significantly positively correlated (*β* = 0.471, *p* < 0.05), GPTID→CA was significantly positively correlated (*β* = 0.382, *p* < 0.05), BP → CA was significantly positively correlated under both intergroup and intragroup levels, (*β* = 0.170, *p* < 0.05; *β* = 0.480, *p* < 0.001), and GPTID→ TB did not significantly affect the relationship (*β* = −0.042, *p* > 0.05, confidence intervals including 0), and hypothesis H1 was not valid.

**Figure 3:**
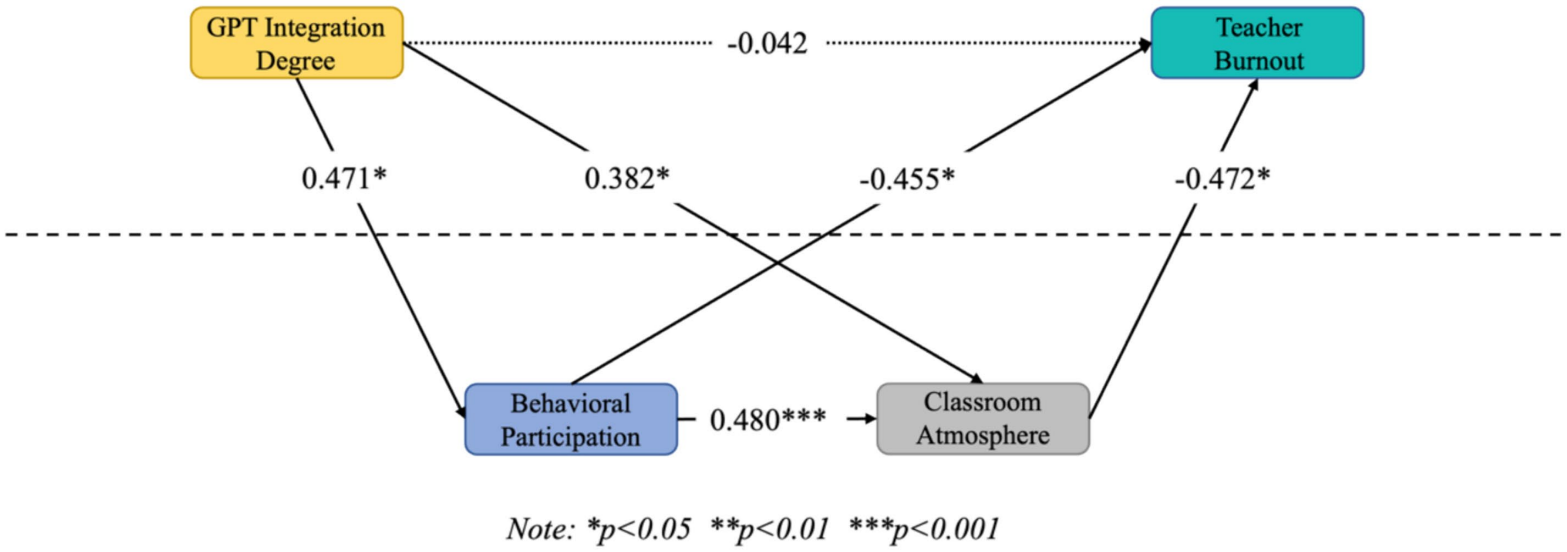
Diagram of model results. *p < 0.05, **p < 0.01, **p < 0.001.

BP was significantly negatively correlated with TB (*β* = −0.455, *p* < 0.05), CA was significantly negatively correlated with TB (*β* = −0.472, *p* < 0.05), GPTID→BP → CA → TB confidence interval excluding 0 chain mediation was significant (*β* = −0.103, *p* < 0.05), GPTID→BP → TB confidence interval excluding 0 mediation effect was significant (*β* = −0.214, *p* < 0.05), GPTID→CA → TB confidence interval does not include 0 mediated effect is significant (*β* = −0.181, *p* < 0.05), hypotheses H2, H3, and H4 are valid, see Figure 3 for details.

## 5 Conclusion and discussion

### 5.1 The direct effect of GPT integration degree on teacher burnout is not valid

Despite the increasing use of GPT in education, the degree of integration did not significantly impact on the direct reduction of teacher burnout. This finding highlights the complexity of integrating GPT into educational settings and its limitations in managing teacher mental health. Teacher burnout is a complex issue influenced by multiple factors, including job stress, lack of resources, job satisfaction, and student behavior. While GPT integration improves technical support and instructional content innovation, it does not directly address the primary stressor in teachers’ work—student engagement and interaction. Insufficient student engagement can lead to poor GPT adoption outcomes, an issue that is currently underappreciated in technology deployment strategies.

First, GPT technology in education is primarily used to improve the quality of instructional content and teaching methods, including creating personalized learning paths and automatically grading assignments. However, authentic learning engagement requires greater interactivity and personal connection, which current technology cannot directly provide. While systems using GPT technology can generate tailored teaching content, this does not guarantee increased student engagement if the content does not captivate or resonate with students. If teachers are not aware of this disconnect, they may persist with ineffective teaching strategies and materials, which may exacerbate feelings of frustration and burnout. Furthermore, the efficacy of educational technology depends on the quality of the support measures it provides (Dowling and Barry, 2020). Integrating GPT technology often requires a degree of technological proficiency on the part of teachers, which in itself can become a source of new stress. If technical training is insufficient or support systems are inadequate, additional technical tasks will increase teachers’ workload. Over time, this burden can lead to increased teacher burnout rather than decreased. Therefore, the provision of adequate and effective technical support is crucial to ensure that the implementation of educational technology achieves the desired pedagogical outcomes.

At the same time, the generalizability of research results across different educational contexts needs to be further explored. Different types of schools may face different challenges and opportunities in the application of GPT. Urban schools usually have better technical equipment and training resources, and teachers may be more willing to integrate GPT. However, the lack of technical infrastructure in rural schools may hinder the integration process and cause teachers to face more technical pressure (Hashem et al., 2024). Different education stages also affect the effectiveness of GPT integration. Students in higher education have stronger self-directed learning abilities, so the effect of GPT on improving the classroom atmosphere is more significant. However, in primary schools, teachers need to devote more energy to managing student behavior, and the integration of GPT may not significantly reduce their workload (Ali et al., 2024). In addition, differences in cultural backgrounds are also important factors affecting the effectiveness of GPT integration. In a cultural context that values teacher-student interaction (such as East Asian cultures), students may rely more on teacher guidance than on adaptive technology, so GPT may have less of an immediate effect on reducing teacher burnout (Mai et al., 2024). In a cultural context that emphasizes student self-directed learning (such as North America, Northern Europe, etc.), GPT technology may be more helpful in reducing teacher workload and improving teaching efficiency. The resources and support systems of different schools also affect the effectiveness of GPT integration. Schools with a well-developed technical support system can provide teachers with more training opportunities and reduce the pressure caused by technological ineptitude. In schools with insufficient technical support, teachers may feel more pressure due to the difficulties encountered in integrating GPT, which in turn affects the degree of burnout. Finally, students’ technological literacy and learning habits also affect the effectiveness of GPT integration. Students with higher technological literacy can better use GPT to improve learning engagement, while students with lower technological literacy may need additional guidance from teachers, which in turn increases the workload of teachers.

### 5.2 Classroom atmosphere plays an important mediating role in GPT integration degree influencing teacher burnout

We can find that GPT, an emerging technology, can greatly enhance students’ internal motivation to learn by providing personalized learning experience, instant feedback mechanism and improving learning efficiency, which makes students’ mentality turn from “I want to learn” to “I want to learn, I want to learn! “, specifically externalized to actively participate in the classroom, creating a proactive classroom atmosphere, thereby reducing teacher burnout. Specifically, the following three aspects can be used to understand the role of classroom atmosphere in bridging the gap between GPT integration degree and teacher burnout. (1) Increase interactivity and participation: By interacting with GPT, students can practice and ask questions in a real-time feedback environment, increasing learning motivation and efficiency, reducing delays and absences caused by teacher distraction, and solving problems and acquiring knowledge in the first time. (2) Customized teaching: GPT not only allows teachers to adjust the teaching progress according to the characteristics of each student, to carry out characteristic teaching, and to reduce the negative emotions caused by a single repetitive work. At the same time, it can also be tailored to the individual, tailor-made learning plans for students, answer questions, improve performance, and create a personalized classroom atmosphere. For example, researchers at Stanford University have used GPT to develop a series of educational tools that personalize student learning, especially in programming and engineering courses. These tools help students understand complex concepts by analyzing student input and providing targeted feedback. In this way, students receive immediate and specific feedback, which not only helps them improve their individual learning, but also strengthens their understanding and mastery of the course, further reinforcing learning outcomes (Aftabi et al., 2024).(3) Improved learning efficiency: With the introduction of GPT as an AI, students can learn in a more challenging and interactive environment, which has been proven to increase student engagement and learning outcomes, while teachers can devote more time and energy to curriculum design and high-value classroom interactions, rather than being physically and mentally exhausted by answering the same questions over and over again. This increased efficiency has a direct impact on the classroom atmosphere, making it more focused and productive, and ultimately reducing teacher workload and burnout (Markel et al., 2023).

### 5.3 GPT integration degree indirectly ordered to influence teacher burnout through behavioral engagement, classroom atmosphere

Rooted in the Chinese tradition, the fundamental task of teachers from ancient times is to “preach, receive and explain,” so education is not a one-man show for teachers, and the ultimate purpose of the teaching profession is ultimately reflected in the students (Chen, 2015). In the field of education, GPT’s high level of integration firstly promotes students’ behavioral engagement directly by providing personalized learning paths and interactive learning platforms. This behavioral engagement effectively enhances student motivation and classroom effectiveness by increasing interactivity and engagement in the classroom. Through automatically generated interactive questions and simulation activities, students are able to explore subject matter content in greater depth, thereby increasing classroom activity and instructional interactivity. And as student behavioral engagement increases, the classroom atmosphere becomes more dynamic and vibrant as a result. This improved classroom atmosphere helps to reduce teacher stress and burnout because teachers are able to observe positive student responses and learning outcomes, which provides positive feedback and a sense of accomplishment. Teachers can thus reduce the one-way knowledge transfer that is common in traditional teaching and instead facilitate and guide the students’ learning process more (Harini, 2023).

However, there are also limitations to the use of GPT, which may affect its long-term effectiveness in educational settings. For example, Albadarin’s study points out that although GPT can generate personalized learning content, there is uncertainty about the quality and accuracy of this content generation, which may lead to students receiving incorrect or insufficient information (Albadarin et al., 2024). In addition, AI-generated feedback is still no substitute for face-to-face communication between teachers and students in terms of emotional support and in-depth interaction, which is particularly obvious in educational and cultural contexts that place a particular emphasis on student-teacher interaction. Teaching with GPT also risks de-professionalizing teachers. Teachers may become over-reliant on AI tools for lesson planning and content delivery, reducing their development of pedagogical skills and strategies. This dependency may in the long term undermine teachers’ autonomy and even leave them feeling overwhelmed when faced with complex teaching situations. For example, research has shown that teachers may feel at a loss when faced with non-standardized problems or students who need emotional support when AI tools take up a large proportion of the classroom (Ali et al., 2024).

In addition, the integration of GPT technology may also lead to issues of technological burden. Teachers need technical training and support systems when using these tools. If this support is inadequate, it can increase teachers’ workload and create new sources of stress (Bettayeb et al., 2024). In educational environments with limited resources, such as some rural schools or areas with scarce educational resources, the technical threshold for integrating GPT is relatively high, which may also exacerbate the imbalance of educational resources. For example, in a computer science course at Case Western Reserve University, the professor used GPT to supplement teaching by generating programming problems in real time and answering programming challenges posed by students. In this way, students can get instant feedback, which motivates them to try more programming exercises. Course feedback shows that the use of GPT significantly improves student engagement, as they discuss problems with professors and classmates more frequently and the classroom atmosphere becomes more active (Cipriano and Alves, 2023). However, the successful application of this technology requires strong technical support and a high level of teacher engagement. Otherwise, it may be difficult to promote it in under-resourced environments.

In summary, although the integration of GPT technology indirectly reduces teacher burnout through behavioral engagement and classroom atmosphere, its use in education also faces challenges such as content quality, changes in the professional role of teachers, and technology burden. Therefore, in the future, when promoting GPT technology, it is necessary to focus on maintaining the professional autonomy of teachers while providing adequate technical support, and to strengthen ethical and educational fairness considerations in the use of technology.

## 6 Shortcomings and prospects

Although this paper addresses the role of GPT integration degree, classroom atmosphere, and behavioral engagement on teacher burnout, there are still some shortcomings that need to be further explored in future studies. Firstly, in terms of the choice of research methods, this study employs quantitative methods, such as structural equation modeling. Future research could consider adopting qualitative analysis methods as the main research method, or alternatively, utilize multiple research methods, such as mixed methods combining qualitative and quantitative methods, in order to enhance the rigor and logical coherence of the article. Secondly, in the process of selecting variables, we selected a limited number of variables that were closely related to the topic of this study. In this process, we inevitably neglected the influence of other factors on teacher burnout, which is limited and subjective. In future studies, it would be beneficial to consider formulating the variables from a more diversified perspective, including factors such as career satisfaction, social support, family responsibility, and so forth. This would enable a more comprehensive and perfect analysis and discussion of the issue.

## Data Availability

The original contributions presented in the study are included in the article/supplementary material, further inquiries can be directed to the corresponding author.

## Appendix

**Table A1:** Questionnaire.

Variant	Subject	Mark
GPT integration degree	I can basically teach myself how to use Chat GPT technology by watching instructional videos and other methods.	1 Strongly disagree 2 Quite disagree 3 Fairly agree 4 Quite agree 5 Strongly agree
	I do not think I’m going to give up on supplementing the use of Chat GPT technology in my teaching for the rest of my life!	1 Strongly disagree 2 Quite disagree 3 Fairly agree 4 Quite agree 5 Strongly agree
	I’m willing to try to explore new features and uses for Chat GPT technology-assisted instruction	1 Strongly disagree 2 Quite disagree 3 Fairly agree 4 Quite agree 5 Strongly agree
	I have experimented with using Chat GPT technology to assist in my teaching tasks at the moment	1 Strongly disagree 2 Quite disagree 3 Fairly agree 4 Quite agree 5 Strongly agree
	I am passionate about Chat GPT technology and would love to learn it!	1 Strongly disagree 2 Quite disagree 3 Fairly agree 4 Quite agree 5 Strongly agree
Behavioral engagement	Classroom teachers using Chat GPT technology to assist with instruction would be more refreshing to me	1 Strongly disagree 2 Quite disagree 3 Fairly agree 4 Quite agree 5 Strongly agree
	I was even more intrigued when I learned that teachers were using Chat GPT as a technology aid.	1 Strongly disagree 2 Quite disagree 3 Fairly agree 4 Quite agree 5 Strongly agree
	When learning that teachers use Chat GPT as a technology-assisted instruction will enhance my classroom engagement ideas	1 Strongly disagree 2 Quite disagree 3 Fairly agree 4 Quite agree 5 Strongly agree
	I will be more proactive in working with teachers to use Chat GPT as a technology aid for instruction	1 Strongly disagree 2 Quite disagree 3 Fairly agree 4 Quite agree 5 Strongly agree
	Teachers using Chat GPT technology in class to assist with instruction would be more likely to pique my interest in learning	1 Strongly disagree 2 Quite disagree 3 Fairly agree 4 Quite agree 5 Strongly agree
Classroom atmosphere	Watching the new form of classroom work done by the instructor using Chat GPT technology made me enjoy the discussion with my classmates even more!	1 Strongly disagree 2 Quite disagree 3 Fairly agree 4 Quite agree 5 Strongly agree
	Having Chat GPT technology in the classroom is more likely to keep me focused	1 Strongly disagree 2 Quite disagree 3 Fairly agree 4 Quite agree 5 Strongly agree
	The addition of Chat GPT technology in the classroom will make me more willing to interact with the teacher	1 Strongly disagree 2 Quite disagree 3 Fairly agree 4 Quite agree 5 Strongly agree
	I feel that my students are more engaged in class when Chat GPT technology is present in their teaching activities.	1 Strongly disagree 2 Quite disagree 3 Fairly agree 4 Quite agree 5 Strongly agree
Teacher burnout	I often get frustrated in my teaching job	1 Strongly disagree 2 Quite disagree 3 Fairly agree 4 Quite agree 5 Strongly agree
	I feel like I’m working too hard.	1 Strongly disagree 2 Quite disagree 3 Fairly agree 4 Quite agree 5 Strongly agree
	I’m afraid this job will make me emotionally numb.	1 Strongly disagree 2 Quite disagree 3 Fairly agree 4 Quite agree 5 Strongly agree
	I felt that when problems arose in the educational process students blamed me for everything that went wrong with them	1 Strongly disagree 2 Quite disagree 3 Fairly agree 4 Quite agree 5 Strongly agree
	Dealing with people in the school (students, other teachers, etc.) stresses me out	1 Strongly disagree 2 Quite disagree 3 Fairly agree 4 Quite agree 5 Strongly agree
	When I wake up in the morning and think about facing a day of teaching, I feel so exhausted	1 Strongly disagree 2 Quite disagree 3 Fairly agree 4 Quite agree 5 Strongly agree
	I do not think I’ll have a better teaching career.	1 Strongly disagree 2 Quite disagree 3 Fairly agree 4 Quite agree 5 Strongly agree
	Many times I feel like I’m in a state of mindlessness at work	1 Strongly disagree 2 Quite disagree 3 Fairly agree 4 Quite agree 5 Strongly agree
